# Letter to the editor: willingness *v*. ability to pay for a universal cost-shared school food programme in Canada

**DOI:** 10.1017/S1368980024001940

**Published:** 2024-10-24

**Authors:** Dana Lee Olstad, Eldon Spackman

**Affiliations:** Department of Community Health Sciences, Cumming School of Medicine, University of Calgary, 3280 Hospital Drive NW, Calgary, AB T2N 4Z6, Canada

**Keywords:** School food programme, Child nutrition, Willingness to pay, Ability to pay

Childhood is a critical period for establishing healthy dietary patterns to support optimal physical and mental health across the life course^(1)^. However, the dietary patterns of children in Canada are poor^(2,3)^, particularly during school hours^(4)^. Offering healthy meals within school meal programmes may be a promising means of improving the quality of children’s dietary intake during school hours^(5,6)^; however, Canada has not yet implemented a federally funded school food programme, although a policy framework was recently released^(7)^.

It is within this context that Datta Gupta et al.^(8)^ sought to examine parents’ and caregivers’ willingness to participate in and pay for a universally offered cost-shared school food programme in Canada. Willingness to pay is an important concept in the behavioural economics literature as it represents the maximum amount individuals are willing to pay for a product or service^(9)^. In the context of school meals, parental willingness to pay may depend upon factors such as the quality of school meals, their household income, food insecurity status, age, gender, ethnicity and many others. In the literature, the most commonly used direct method to elicit willingness to pay is contingent valuation, in which an individual is asked the maximum they would be willing to pay for a particular service or good. However, many consider direct methods to be unreliable and prefer indirect methods such as conjoint analysis or discrete choice analysis^(10)^.

We write to express several concerns with the methods Datta Gupta et al.^(8)^ used to elicit willingness to pay for a school food programme using the contingent valuation method. In particular, the article does not uphold established scientific standards regarding reporting practices and ensuring that measurement tools are valid for their intended purpose.

In the methods section of their article, the authors describe their sampling strategy, outline the topics addressed in their survey, indicate that they assessed ‘willingness to pay’ and provide details of their models and analytic strategy. However, the specific questions that were used to assess willingness to pay are not stated in the methods, although they are available in the first author’s thesis^(11)^. First, participants were given a description of the type of universal cost-shared meal programme that the investigators wished them to consider—this first part alone was nicely detailed in the published paper. Participants were subsequently asked ‘Would you want your child(ren) to participate in such a program?’ Those who responded affirmatively were then asked ‘Would you be able to afford a daily payment of $4·00 CAD per child (about $750 per school year or $75 per month)?’ It was this question, with subsequent bids of $8·00 CAD and $2·00 CAD per child, which formed the crux of Datta Gupta et al.’s analysis of willingness to pay. A final question asked ‘Can you tell us what would be the maximum amount you would be able to pay daily per child for a program like this?’

Herein lies the problem, as although Datta Gupta et al.^(8)^ purport to have assessed *willingness to pay* and hence *demand* for a school food programme, it is evident that based on the questions asked (i.e., Would you be *able to afford*…), they actually assessed *ability to pay* and hence the *affordability* of a school food programme. Although *willingness* and *ability to pay* are related, they are nevertheless distinct concepts. For instance, we (DLO and ES) can *afford to buy* cigarettes and illicit drugs; however, we are not *willing* to do so. Our ability to pay for these items reflects whether we have the financial resources to purchase these products, whereas our willingness to pay for them reflects our actual demand for them. In this case, our demand for these products is zero because we are not willing to pay for them, although we are able to afford them. For some goods, such as a life-saving surgery for a family member, we may not be able to afford to pay for the surgery, but we may be willing to pay for it by going into debt. Again, the concepts of willingness and ability to pay are related but distinct.

To be clear, the problem is not that Datta Gupta et al.^(8)^ assessed ability rather than willingness to pay for a school food programme—as both are important concepts to assess in relation to school food programmes. Rather, the problem is that the authors assessed *ability to pay* and hence the affordability of school meals, but they claim to have assessed *willingness to pay* and hence demand for school meals. If the authors aimed to assess demand for a school food programme in Canada, as indicated by their objectives, why did they not simply ask ‘Would you be *willing to pay* $4·00 CAD per child (about $750 per school year or $75 per month)?’

To summarise, our main concerns with this article are therefore three-fold. First, for the sake of transparency, verification, and reproducibility, the questions that participants were asked should have been included in the methods section of the paper, or at minimum accurately paraphrased (i.e., the paper states that participants were asked about willingness to pay when in fact they were asked about ability to pay) or included in the supplemental files. This is just good scientific practice—accurately and thoroughly documenting what was done. Second, survey questions should demonstrate content and construct validity. In this case, the question on willingness to pay did not assess what the authors purported that it did and thus lacked both content and construct validity. Third and most importantly, the entire paper is framed as being about willingness to pay and demand for a universal cost-shared school food programme in Canada. Framing the paper and its findings in this way has the potential to misinform policy, given that the authors did not actually assess willingness to pay for a school food programme. Policymakers often turn to the academic literature to inform evidence-based policy, and indeed, the authors themselves state that their findings can inform the design of a universal school food programme in Canada. Were policymakers to take Datta Gupta et al.’s findings at face value, they would in fact be basing their policy on incorrect data.

We approached the authors and asked them to publish a correction. However, they maintained that there is no universally agreed method to assess willingness to pay, that others have assessed willingness to pay using similar questions (although they did not provide any references to substantiate this) and that the Saskatoon Public Schools Division had final say over the wording of the survey questions. However, if others have used similarly worded questions, surely the fact that others have made the same error does not justify continuing this practice? Moreover, upon reviewing the papers cited by Datta Gupta et al. in relation to willingness to pay, we noted that the papers they cited correctly asked about *willingness* not *ability* to pay. For example, Kesztyus et al.^(12)^ asked ‘Would you be *willing to pay* for the prevention of overweight and obesity in children?’ and ‘How much would you be *willing to pay* in addition to the health insurance contributions you already have to pay?’ Similarly, Cerda et al.^(13)^ asked ‘Are you willing to pay US $*bid*?’ Moreover, if the Saskatoon Public Schools Division was responsible for the final wording of the questions, why did the authors not simply reorient their paper around the concept of ability to pay for a school food programme, rather than persisting in claiming to have assessed willingness to pay for such a programme? We therefore urge the authors to correct the scientific record to avoid misleading future research and policy.
